# Automatized protocol and interface to simulate QM/MM time‐resolved transient absorption at TD‐DFT level with COBRAMM

**DOI:** 10.1002/jcc.26966

**Published:** 2022-07-11

**Authors:** Davide Avagliano, Matteo Bonfanti, Artur Nenov, Marco Garavelli

**Affiliations:** ^1^ Dipartimento di Chimica Industriale "Toso Montanari" Università degli Studi di Bologna Bologna Italy; ^2^ Fondazione Human Technopole ‐ Viale Rita Levi‐Montalcini, 1 ‐ Area MIND – Cargo 6 ‐ 20157 Milano Italy

**Keywords:** QM/MM, TD‐DFT, trajectory surface hopping, transient absorption, ultrafast spectroscopy

## Abstract

We present a series of new implementations that we recently introduced in COBRAMM, the open‐source academic software developed in our group. The goal of these implementations is to offer an automatized workflow and interface to simulate time‐resolved transient absorption (TA) spectra of medium‐to‐big chromophore embedded in a complex environment. Therefore, the excited states absorption and the stimulated emission are simulated along nonadiabatic dynamics performed with trajectory surface hopping. The possibility of treating systems from medium to big size is given by the use of time‐dependent density functional theory (TD‐DFT) and the presence of the environment is taken into account employing a hybrid quantum mechanics/molecular mechanics (QM/MM) scheme. The full implementation includes a series of auxiliary scripts to properly setup the QM/MM system, the calculation of the wavefunction overlap along the dynamics for the propagation, the evaluation of the transition dipole moment at linear response TD‐DFT level, and scripts to setup, run and analyze the TA from an ensemble of trajectories. Altogether, we believe that our implementation will open the door to the easily simulate the time‐resolved TA of systems so far computationally inaccessible.

## Graphical abstract

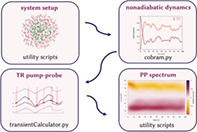

Schematic overview of the protocol implemented in COBRAMM to simulate transient absorption

## 1 INTRODUCTION

**FIGURE 1:**
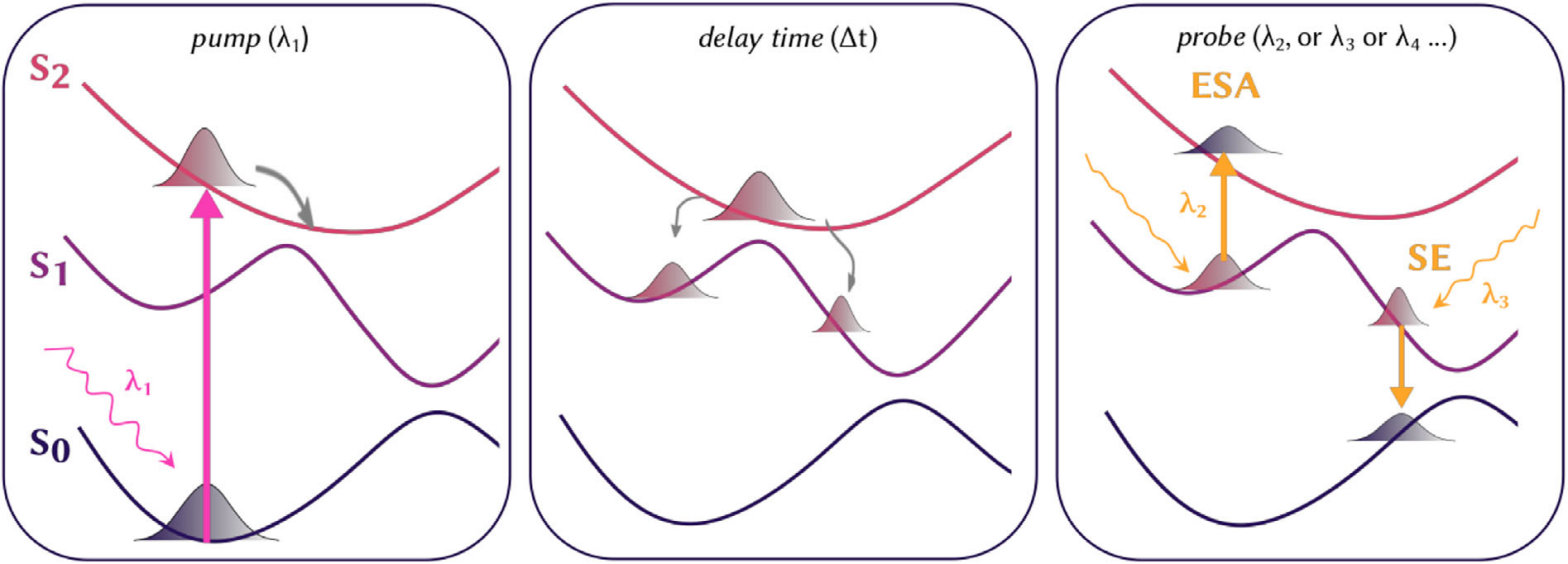
Schematic representation of a transient absorption experiment and of the signals simulated in this work: (A) a pump prob *λ*_1_ excited the system to an excited state and the created wavepacket starts propagating along this potential energy surface (PES); (B) during a certain delay time the wavepacket can split and decay to a low‐lying electronic state; (C) after that delay time a second probe pulse *λ*_2_ can promote the system to a different excited states or induce the relaxation to the ground states and stimulate the emission.

Transient absorption (TA) spectroscopy is a crucial technique to deeply understand the ultrafast dynamics of photoactive systems and can offer a temporal resolution in the order of the femtosecond (fs) time scale.^1^, ^2^, ^3^, ^4^, ^5^, ^6^, ^7^ The basic scheme of an experiment is the following (Figure 1): a wavepacket is initially created on an excited state potential energy surface (PES) by a pump pulse and it starts to propagate along this surface. During its evolution in time, the system can continue propagating on the current PES or relax to a lower‐lying excited state through a nonradiative decay. This dynamics can be probed by a second pulse that additionally hits the excited system after a certain delay time *t*. If the incident probe photon covers the UV/vis spectrum as well, the wavepacket can be promoted to a higher‐lying excited state, the so‐called excited state absorption (ESA). Alternatively, the probe pulse can induce the relaxation to the ground state through a radiative decay, the so‐called stimulated emission (SE). A pump‐probe (PP) spectrum is normally recorded with a first measurement performed with the pump turned off and only the probe beam hitting the system, followed by a second measurement with the pump on. In the first measurement only ground state populations is probed, while in the second one part of the total population is on the excited state. The difference in intensity between the ground state absorption in the two measurements gives rise to the so‐called ground state bleaching, which corresponds to the depletion of the ground state population due to the pump pulse.

Despite the powerfulness of the technique, a computational support is often required to confirm and rationalize the time evolution of the system and the chemical and physical nature of the features giving rise to the signals in the TA spectra.^8^ Many approaches have been reported to simulate such signals with varying compromises between accuracy and cost, documenting an immense interest of the computational photochemistry community to these kinds of simulations and the importance of developing tools in this field.^9^, ^10^, ^11^, ^12^, ^13^, ^14^, ^15^, ^16^, ^17^, ^18^, ^19^ The most rigorous and accurate way to compute TA signal is based on quantum dynamics (QD) simulations, which allow to explicitly consider the electric field of the pulses in the simulations, to vary their strength, temporal and spectral profiles, and other parameters.^18^, ^20^ QD simulations are usually performed on a potential energy grid and the cost associated with pre‐computing these grids, exponential increasing with the number of degrees of freedom (DOF), limits their applicability to two or three DOFs.^21^ A way to consider all the DOFs in the simulation of the optical response of the system is to parametrize a model Hamiltonian in the harmonic approximation (i.e., multidimensional uncoupled displaced harmonic potentials). In the weak field impulsive regime and under the approximation of adiabatic dynamics, the TA signal becomes proportional to the third‐order nonlinear response of the system to the field‐matter interaction.^22^ In this framework, exact analytical equations can be derived. Electronic population dynamics and decoherence can be introduced phenomenologically. Multiconfiguration time‐dependent Hartree represents a powerful way to perform QD on model Hamiltonians with up to tens of DOFs, thereby explicitly considering electric fields and non‐adiabatic effects.^23^ Approaches based on semi‐classical and mixed quantum‐classical trajectories represent a good balance between accuracy and computational efficiency.^24^, ^25^, ^26^, ^27^, ^28^ Trajectory‐based methods allow to consider all DOFs without the limitation of the harmonic approximation, thus generalizing the dynamics to arbitrary systems. Non‐adiabatic effects can be included at various levels of sophistication. Trajectory surface hopping (TSH) is a popular method to simulate nonadiabatic dynamics based on the propagation of independent trajectories that evolve on potential energy surfaces calculated on‐the‐fly that drive the nuclear motion.^29^ The drawback of TSH is that the nuclei follow classical laws of motion, so that the density matrix is not defined which prevents the calculation of quantities directly related to electronic coherences. Subotnik and co‐workers derived an expression for the density matrix consistent with the TSH method, which allowed them to develop a protocol for simulating TA in the impulsive limit which gives excellent results within the short time approximation.^30^ The elevated quality comes at the expense of needing to launch a new propagation of the swarm of trajectories on each electronic state coupled to the photoactive states through the probe pulse at each delay time *t* for an interval τ. More often, a more radical approximation is made, considering only the vertical energy gap between the photoactive state and the remaining states at each delay time *t*, termed Condon approximation, thus assuming instantaneous dephasing. This approach has been used to successfully reproduce TA spectra using wavefunction methods for the description of the excited states.^9^, ^10^, ^12^, ^31^ Recently, Domcke and coworkers reported a TSH compatible protocol within the Condon approximation which accounts for the finite duration and spectral shape of the pump and probe.^9^

In this work, we want to introduce a series of tools implemented in the open‐source academic software developed in our group, Cobramm is Optimized in Bologna to Run Ab‐initio and Molecular‐Mechanics calculations (COBRAMM)^32^, ^33^ that automatize the whole workflow necessary to simulate a TA spectrum, from building the initial system, through simulating the non‐adiabatic dynamics with TSH, until the calculation and convolution of the time‐resolved TA spectrum. Although this protocol can be applied for molecules in the gas phase and to any level of theory desired by the user, we here want to emphasize two possibilities that our implementation offers to the community, namely the explicit inclusion of solvent and environmental effects through a mixed quantum mechanical/molecular mechanics (QM/MM) approach,^34^ and the applicability of the workflow to medium‐to‐large chromophores, thanks to the present extension of the protocol to the time‐dependent density functional theory (TD‐DFT).^35^ The present tool offers the opportunity of studying the TA of systems that would otherwise be too computationally demanding. The article is organized as followed: first of all, the theoretical background needed in all the steps of the protocol is revised; then the details of the new implementations required in COBRAMM are listed; finally the TA spectrum of 4‐(N,N‐dimethylamine)‐benzonitrile (DMABN) is presented as an exemplary application, as its ultrafast nonadiabatic dynamics is well‐known and its TA spectrum has recently been characterized computationally with a different method,^31^ and thus, DMABN represents a perfect test case for our implementations. Particular emphasis will be put on the post‐processing tools developed, which allow the convolution of the PP spectra in the energy and time domain, the application of different polarization combinations and the decomposition of the signal in single transitions contributions.

## 2 THEORY AND METHODS

### 2.1 QM/MM and DFT in COBRAMM

The QM/MM scheme^34^, ^36^ implemented in COBRAMM has been widely reported and explained in literature.^32^, ^33^, ^37^, ^38^ However, we will briefly summarize here the main features of the approach. COBRAMM collects the energy of the QM (*E*_QM_) and MM (*E*_MM(tot)_ and *E*_MM(QM)_) part through its own interfaces to Molcas^39^ and OpenMolcas,^40^ Molpro^41^ and Gaussian^42^ for the QM part, and to AMBER^43^ for the MM one. Having obtained the quantities needed by the third‐party software, it calculates the QM/MM energy with a subtractive scheme:

(1) $$E_{\mathrm{QM}/\mathrm{MM}}=E_{\mathrm{QM}}+E_{\mathrm{MM}\left(\mathrm{tot}\right)}-E_{\mathrm{MM}\left(\mathrm{QM}\right)}\;$$

The evaluation of the electrostatic interaction between the QM and the MM part is enhanced by the inclusion of the electrostatic embedding term, which allows the polarization of the QM part by the inclusion of the MM charges in the Hamiltonian adopted. Additionally, in case of excited states optimization, minimum energy path exploration or nonadiabatic dynamics, the influence of the QM density on the point charges is considered, with a state‐specific contribution being added to the gradient of the point charges. In the case where the QM‐MM boundary region involves covalent bonds, a link‐atom approach is used. The delicate issue of treating boundary regions and the possibly resulting over‐polarization is automatically handled by COBRAMM internally by charge repartitions, as explained in detail in the original publications.^32^, ^33^ In order to treat long‐range electrostatic effects in QM/MM calculations, Ewald‐like approaches^44^ or continuum models^45^ might be used. COBRAMM takes advantages of the subtractive scheme in Equation (1) to treat QM/MM electrostatic in a non‐periodic system: In an ONIOM‐like scheme,^46^ the QM part is called high‐layer (H), while the MM part is subdivided in two regions, medium‐layer (M) and low‐layer (L). The energy of the atoms of both M and L are calculated at MM level and their point charges included in the QM Hamiltonian, but M layer atoms are allowed to move in geometry optimization and dynamics, while the L is kept frozen in order to ensure a constant outer potential and the stability of the solvation created around the H layer. MM calculations are performed through the COBRAMM interface to AMBER that has been rewritten in an object‐oriented way, which enhances its efficiency and applicability, and it allows to calculate energies and gradients at the MM level, as well as to exploit more capabilities of the software, like parametrization or setup of the system. Similarly, TD‐DFT calculations can be carried out through the internal interface to Gaussian that has been fully rewritten as well. A high flexibility is left to the user to exploit all the functionalities of the powerful QM software. In the context of TA spectrum calculations, it is important to use an appropriate functional for the excited states calculation, and to perform the calculation with the Tamm‐Dancoff approximation (TDA), in order to exclude the de‐excitation contributions, for the evaluation of the transition dipole moment (TDM) between excited states (as will be explained in more details in section 3.3). To escape these intrinsic pitfalls of TD‐DFT, the setup is automatized in the auxiliary scripts provided by COBRAMM. Generally, TD‐DFT can be used as a black‐box method, but the awareness of the system under studying is fundamental to choose simulation parameters needed to obtain reliable results. In this regard, high freedom to modify functional, basis set, number of roots and other parameters throughout the preparation steps is guaranteed to the users. The user should be always be aware of the limitations of the method, like the impossibility of describing double excitations within the linear response and adiabatic approximation in TD‐DFT,^47^ whose signature will be missing in the TR spectrum calculated at this level of theory.

### 2.2 Trajectory surface hopping

The great popularity of TSH^48^, ^49^ can be attributed to the approximations intrinsic to the method, which make it easily applicable to study nonadiabatic dynamics of medium‐to‐large size chromophores, otherwise impossible to study, with still a reliable accuracy,^50^ also thanks to the validation and application of TD‐DFT as ab initio method to calculate electronic energies.^51^, ^52^ TSH mimics the motion of a wavepacket along the excited states PES by a swarm of independent classical trajectories that can hop between excited states in order to simulate a decay to a different excited state. The nuclei are moved classically, driven by the electronic propagation obtained by the electronic time‐dependent Schrödinger equation, solved by any possible quantum method. The nonadiabatic event, represented as an instantaneous and finite transition from one state to another, is stochastic, based on a probability algorithm. In the fewest‐switches‐surface‐hopping (FSSH) algorithm, the probability (*P*_*j*_) to hop from a state *j* to another state is given by the electronic coefficients of the wavefunction:

(2) $$P_{j->}\left(t,t+∆t\right)=\frac{{\left|c_{j}\left(t\right)\right|}^{2}-{\left|c_{j}\left(t+∆t\right)\right|}^{2}}{{\left|c_{j}\left(t\right)\right|}^{2}}\;$$

where the time evolution of the electronic coefficients is given by:

(3) $$\frac{\partial c_{j}\left(t\right)}{\partial t}=-\sum_{i}c_{i}\left(t\right)\left[\frac{i}{\hbar }H_{\mathrm{ji}}+T_{\mathrm{ji}}\right]\;$$

Beside the energies of state *i* and *j*, the hopping between them is driven by the time‐derivative coupling (TDC) *T*_*ji*_ which is the dot product of the velocity and the nonadiabatic coupling (NAC) vector. The NAC can be computed at each step along the dynamics or can be approximated by computing the wavefunction overlap (WFO) of the two electronic states involved.^53^ This can be done in order to speed up the calculation time, or if the NAC vector calculation is not available in a specific software or not possible within the framework of a QM method. This is the case of TD‐DFT and two methods to calculate the WFO implemented in COBRAMM will be introduced in section 3.2. The FSSH algorithm was already implemented in COBRAMM and includes the energy‐based decoherence correction scheme,^54^ the velocity‐Verlet integration,^55^ the kinetic energy rescaling to preserve the total energy after a hop, and many other simulation parameters that can be set by the user. For example, in TD‐DFT trajectories, an induced hop to the ground state can be included, in order to approximately describe a relaxation to *S*_0,_ which cannot be properly describe with linear response TD‐DFT within the adiabatic approximation.^47^ This hop is based on energy gap between S_1_ and S_0_, at the choice of the user.

### 2.3 Time‐resolved pump‐probe spectrum

The time‐resolved TA spectrum can be computed from an ensemble of trajectories independently propagated that sample the evolution of the wavepacket along the potential energy surfaces.^9^, ^12^, ^13^, ^31^ Considering a single trajectory evolving on the PES of an active state *a*, at each time step the TA spectrum can be obtained by calculating vertical excitations from state *a*. The SE is computed considering the oscillator strength (*f*) with respect to the ground state, while the ESAs are evaluated considering *f* of transitions between state *a* and the higher‐laying excited states calculated. The spectrum at a certain time *t* is convoluted with a sum of Gaussian functions centered at the ESA and SE energies and with an intensity proportional to *f* of the transition. The intensity of the ESA from the state *a* to an excited state *e* is given by:

(4) $$I_{\mathrm{ESA}}\left(E,t\right)=\sum_{e}{\left|f_{a->e}\left(R\left(t\right)\right)\right|}^{2}\frac{1}{\text{FWHM}\sqrt{2\pi }}\exp\left(\frac{{\left(E_{e}\left(R\left(t\right)\right)-E_{a}\left(R\left(t\right)\right)-E\right)}^{2}}{2{\text{FWHM}}^{2}}\right)\;$$

and, analogously, the SE from state *a* to the ground state (*gs*) is given by:

(5) $$I_{\mathrm{SE}}\left(E,t\right)=-{\left|f_{a->\mathrm{gs}}\left(R\left(t\right)\right)\right|}^{2}\left(\frac{1}{\text{FWHM}\sqrt{2\pi }}\exp\left(\frac{{\left(E_{\mathrm{gs}}\left(R\left(t\right)\right)-E_{a}\left(R\left(t\right)\right)-E\right)}^{2}}{2{\text{FWHM}}^{2}}\right)\right)\;$$

In principle, SE to any state below the “active” state *a* in which the system evolves at the time of the interaction with the probe pulse could be observed/simulated. However, these transitions fall very often below the detection limit of the experimental apparatus and it is commonly observed and simulated only the SE to the ground state.

Summing over all the trajectories we can obtain the TA for a certain time and convoluting over all the time steps *t*, we obtain the final TA as:

(6) $$I\left(E,t\right)=\frac{1}{N_{\mathrm{trj}}}\sum_{k}^{N_{\mathrm{trj}}}I_{\mathrm{ESA},k}\left(t\right)+I_{\mathrm{SE},k}\left(t\right)\;$$

Regarding the convolution with respect to an experimentally recorded spectrum, some considerations regarding our implementation need to be pointed out: (i) the Gaussian line shape used to convolute the spectrum is artificially broadened by FWHM to smooth the signal, removing the noise; (ii) three possible arrangements of the pump and probe pulses are considered.^56^ The first one is at the so‐called “magic angle,” for which the intensity of the signal is assumed to be only dependent on the magnitude of the TDM and independent from the angle between the dipole moments and the angle between the pump and probe pulse. Other two possible arrangements considered are the parallel and the orthogonal. In these two cases, the intensity of the signal is scaled by a factor A that corresponds to

(7) $$A\left({\mathrm{TDM}}_{i},{\mathrm{TDM}}_{j}\right)=\frac{1}{15}\left[1+2\cos^{2}\theta_{{\mathrm{TDM}}_{i},{\mathrm{TDM}}_{j}}\right]\;$$

in case of parallel arrangement and to

(8) $$A\left({\mathrm{TDM}}_{i},{\mathrm{TDM}}_{j}\right)=\frac{1}{15}\left[2-\cos^{2}\theta_{{\mathrm{TDM}}_{i},{\mathrm{TDM}}_{j}}\right]\;$$

in case of orthogonal arrangement, where TDM_i_ represents the transition dipole moment vector of the transition promoted by the pump at *t*_0_ and TDM_j_ the one promoted by the probe at time *t* and *θ* the angle between them. That means that a parallel arrangement leads to a signal three times more intense for transition that have TDM parallel to the one generated by the pump pulse, while the one orthogonal to it will be two times more intense for orthogonal arrangement, and in general indicates a modulation of the relative intensities of the signals; (iii) an additional broadening in the time domain as

(9) $$I\left(E,t_{i}\right)=\int_{-\infty }^{+\infty }I\left(E,\tau \right)\exp\left(-\frac{\left(\tau -t_{i}\right)}{2{\text{FWHM}}^{2}}\right)\mathrm{d\tau}\;$$

is applied to account for finite pulse duration; (iv) white light is assumed for the probe pulse, which means that the pulse covers the whole spectral window of interest.

## 3 NEW IMPLEMENTATIONS IN COBRAMM

### 3.1 Preparation and analysis scripts for nonadiabatic dynamics

We implemented a new series of auxiliary scrips in COBRAMM that help the user prepare the system step‐by‐step, and analyze the results of the simulation. These python scripts are added to the already present system‐preparation tool (*cobramm.prep*), with the advantage of being divided according to the individual task to be performed. This allows to use all of them in serial or independently for particular tasks needed by the user.

An extremely important, yet delicate, task is the generation of meaningful initial conditions used to propagate the trajectories.^57^, ^58^ This aspect becomes even more sensitive in case of QM/MM setups.^59^ In COBRAMM, we tackle this task in two ways, depending on the complexity of the system.

#### 3.1.1 Isolated chromophores in solution

This task is fully covered by new python scripts. Starting from an isolated geometry of a chromophore, that might be one or more organic molecules, this is solvated with a box of solvent molecules and the whole system is minimized, heated, and equilibrated at the MM level with a standard procedure, as described in section 4.1, using General Amber Force Field^60^ (GAFF) for the chromophores. Additionally, charges computed by the user can be parsed to the setup script, in order to refine the parametrization or for example describe the charge distribution of an excited state. In this case, the chromophore can be kept frozen to equilibrate the solvent around the charge distribution of the excited state. At the end of the simulation, energy minimization, heating and pressure equilibration profiles are saved in pdf format to be easily visualized by the user. A second script creates a droplet of solvent molecules and partitions the system in high, medium, and low layer. This is important in the next step, where the H and M layer are optimized by COBRAMM at QM/MM level, with the QM level of choice of the user. Having calculated the frequencies of the QM part at the obtained minimum, another script generates a distribution of positions and momenta for each normal mode of the chromophore based on a Wigner probability distribution.^61^ The solvent molecules included in the M layer are a‐posteriori equilibrated classically around these H‐layer geometries obtained and kept frozen. This is done by a last preparation script that also creates the input file to propagate TSH nonadiabatic dynamics from these starting geometries and momenta.

#### 3.1.2 Chromophore embedded in a complex environment

More complicated cases than isolated organic chromophores in solution require more attention of the user. These systems include for example biological structures, molecular aggregates, transition metal complexes or any other non‐trivial environment surrounding the molecular units of interest. In these cases, the building of the system, the force field parametrization and an eventual conformational sampling through classical molecular mechanics are responsibility of the user. Indeed, a full automatization of such process for complex systems would lack of the required chemical and technical intuition and experience of the user. However, we offer a procedural support through this preparation step through the already existing *cobramm.prep* script, which requires minimal input by the user and allows for unconstrained layer definition.^32^

After a proper ensemble of initial conditions for TSH dynamics has been generated, the trajectories can be propagated by COBRAMM. Once the simulations are terminated, the results can be analyzed by additional python scripts. Total kinetic and potential energies, occupation numbers, and wavefunction amplitudes can be extracted and saved for the user, as well as fractions of trajectories/state and relative populations can be calculated and stored.

### 3.2 Built‐in calculation of wave function overlaps

As suggested by Hammes–Schiffer and Tully, the nonadiabatic coupling between two states can be reliably approximated by the overlap between the wavefunction of these states.^53^ In this way, the TDC can be expressed as:

(10) $$T_{\mathrm{ij}}=\left\langle \Psi_{i}\left(r,t\right)|\frac{d}{\mathrm{dt}}\Psi_{j}\left(r,t\right)\right\rangle \;$$

In COBRAMM, the TDC is approximated to finite difference and computed from the anti‐symmetrized projection, using the overlap matrix at three consecutive points

(11) $$T_{\mathrm{ij}}=\begin{array}{l} \frac{1}{4∆t}[3*\left(\left\langle \Psi_{i}\left(t\right)|\Psi_{j}\left(t-∆t\right)\right\rangle -\left\langle \Psi_{j}\left(t\right)|\Psi_{i}\left(t-∆t\right)\right\rangle \right)\\ -\left(\left\langle \Psi_{i}\left(t-∆t\right)|\Psi_{j}\left(t-2∆t\right)\right\rangle -\left\langle \Psi_{j}\left(t-∆t\right)|\Psi_{i}\left(t-2∆t\right)\right\rangle \right)] \end{array}$$

with only the first term computed in the first time step. Different algorithms have been developed with the goal of calculating WFO^62^, ^63^ to be applied for the calculation of TDCs. We firstly implemented in COBRAMM an internal routine that calculates the WFO between the wavefunction between two consecutive steps along the dynamics rigorously. This implies computing the overlap between all the Slater determinants and can require a non‐negligible amount of time in comparison to the time required to run the other routines during a time step.

We then implemented a second, faster, way of computing WFO that relies on transformational sub‐matrices of occupied and virtual orbitals.

In TD‐DFT within the Tamm‐Dancoff approximation^64^ (TDA), the wavefunction Ψ_*i*_ (*r*) of the *i*th electronic state at position r can be written as:

(12) $$\Psi_{i}\left(r\right)=\sum_{a}^{n}\sum_{p}^{m}C_{\mathrm{ia}}^{p}\Phi_{a}^{p}\left(r\right)\;$$

with $C_{\mathrm{ia}}^{r}$ and $\Phi_{a}^{p}$ being the CI coefficients of state i and are the determinants associated with each single excitation from occupied orbital a to virtual orbital p, respectively. With this definition the overlap term

(13) $$\left\langle \Psi_{i}\left(r\right)|\Psi_{j}\left(r^{\prime }\right)\right\rangle \;$$

between states i and j at two different geometries r and $r^{\prime }$ becomes

(14) $$\left\langle \Psi_{i}\left(r\right)|\Psi_{j}\left(r^{\prime }\right)\right\rangle =\sum_{a}^{n}\sum_{b}^{n}\sum_{p}^{m}\sum_{s}^{m}\overset{p}{\underset{\mathrm{ia}}{C}}\overset{p}{\underset{\mathrm{jb}}{C}}\left\langle \Phi_{a}^{p}\left(r\right)|\Phi_{b}^{p}\left(r^{\prime }\right)\right\rangle \;$$

where

(15) $$\Phi_{a}^{p}\left(r\right)=|\psi_{1}\left(r\right),\ldots ,\psi_{a-1}\left(r\right),\psi_{p}\left(r\right),\psi_{a+1}\left(r\right),\ldots ,\psi_{n}\left(r\right)\rangle \;$$

with $\psi_{p}\left(r\right)$ being the p‐th molecular orbital (MO). The determinant overlap term

(16) $$\left\langle \Phi_{a}^{p}\left(r\right)|\Phi_{b}^{s}\left(r^{\prime }\right)\right\rangle \;$$

is equal to determinant of the MO overlap matrix $S_{a,b}^{p,s}$

(17) $$\begin{array}{rcl} \left\langle \Phi_{a}^{p}\left(r\right)|\Phi_{b}^{s}\left(r^{\prime }\right)\right\rangle & \;= & \;\det S_{a,b}^{p,s}\left(r,r^{\prime }\right)\\ & \;= & \;\det[\left(\begin{array}{c} \left\langle \psi_{1}\left(r\right)\right|\\ \vdots \\ \left\langle \psi_{a-1}\left(r\right)\right|\\ \left\langle \psi_{p}\left(r\right)\right|\\ \left\langle \psi_{a+1}\left(r\right)\right|\\ \vdots \\ \left\langle \psi_{n}\left(r\right)\right| \end{array}\right)⨂(\left|\psi_{1}\left(r^{\prime }\right)\right\rangle ,\ldots ,\left|\psi_{b-1}\left(r^{\prime }\right)\right\rangle ,\left|\psi_{s}\left(r^{\prime }\right)\right\rangle ,\\ & & \left|\;\psi_{b+1}\left(r^{\prime }\right)\right\rangle ,\ldots ,|\psi_{n}\left(r^{\prime }\right)\rangle )] \end{array}$$

$S_{a,b}^{p,s}$ is the overlap matrix of the lowest occupied orbitals (dimension n×n), where the *a*th row and *b*th column have been replaced with the *p*th row and *s*th column of the full MO overlap matrix (dimension ($n+m)\times \left(n+m\right)$), respectively, and the *a,b*th element has been replaced by the *p,s*th elements.

The MO overlap matrix at the same geometry r has the form of a unitary matrix (due to the orthonormalization of the MOs), thus

(18) $$S_{a,b}^{p,s}\left(r,r\right)=\delta_{\mathrm{ab}}\delta_{\mathrm{ps}}I\;$$

This is not the case for different geometries r and $r^{\prime }$. An unitary transformation of the MOs (i.e., a rotation that satisfies the orthonormality requirement) does not change the expectation values such as the energy. Making the assumption that the occupied and virtual subsets of MOs present complete orbital sets, it implies that there exist unitary transformation matrices $U_{\mathrm{occ}}$ (dimension n×n) and $U_{\text{virt}}$ (dimension m×m) which rotate the subsets of the occupied and virtual orbitals at geometry $r^{\prime }$ to maximize the overlap with the corresponding subsets at geometry r. The transformational matrices are the inverse of the $S_{\mathrm{occ},\mathrm{occ}}$ and $S_{\text{virt},\text{virt}}$ blocks of the full MO overlap matrix.

(19) $$U_{\mathrm{occ}}=S_{\mathrm{occ},\mathrm{occ}}^{-1}\;$$

(20) $$U_{\text{virt}}=S_{\text{virt},\text{virt}}^{-1}$$

Essentially, we neglect the off‐diagonal blocks of the full MO overlap matrix $S_{\mathrm{occ},\text{virt}}$ and $S_{\text{virt},\mathrm{occ}}$. This approximation holds as long as the ground and excited state potential energy surfaces are energetically separated, implying a large energy gap between the occupied and virtual orbitals and, as a consequence, negligible mixing. The approximation breaks at crossings between the ground and first excited state, that is, *S*_1_/*S*_0_ conical intersection (CoIns) where the frontier orbitals are nearly degenerate and mix strongly. However, this does not present a limitation of the presented method, as single reference methods exhibit convergence problems in the vicinity of the *S*_1_/*S*_0_ CoIns and the dynamics are interrupted in case such a CoIns region is approached. In fact, the off‐diagonal elements can be used as a diagnostic tool to assess the validity of the approximation.

(21) $$B_{a}^{\text{virt}}=\sum_{p}^{m}{\left\langle \psi_{a}\left(r\right)|\psi_{p}\left(r^{\prime }\right)\right\rangle }^{2}\;$$

$B_{a}^{\text{virt}}$ denotes the projection of the occupied orbital *a* at geometry *r* on the subset of virtual orbitals at geometry $r^{\prime }$.

Since the single electron excitation requires to exchange an MO from the occupied subset with an MO from the virtual subset, the CI coefficients at geometry $r^{\prime }$ are modified by the transformation via the expression

(22) $$C_{i}^{\prime }=U_{\mathrm{occ}}^{T}C_{i}U_{\text{virt}}=S_{\mathrm{occ},\mathrm{occ}}^{-T}C_{i}S_{\text{virt},\text{virt}}^{-1}=S_{\mathrm{occ},\mathrm{occ}}C_{i}S_{\text{virt},\text{virt}}^{-1}\;$$

where $C_{i}$ is defined as the matrix of CI coefficients of dimension n×m. Since $U_{\mathrm{occ}}$ is unitary matrix, the transpose of its inverse gives the $U_{\mathrm{occ}}$ matrix itself. Thus, only the inverse of the MO overlap matrix of the virtual orbitals needs to be computed. In practice, since the occupied and virtual blocks are not complete, the two expressions (whether using $S_{\mathrm{occ},\mathrm{occ}}^{-T}$ or $S_{\mathrm{occ},\mathrm{occ}}$) give slightly different results which are related to the quality of the approximation of completeness.

With the above MO transformation at geometry $r^{\prime }$ it is achieved that the full MO overlap matrix becomes a unitary matrix

(23) $$S_{a,b}^{p,s}\left(r,r^{\prime }\right)=\delta_{\mathrm{ab}}\delta_{\mathrm{rs}}I\;$$

Consequently, the determinant overlap term $\left\langle \Phi_{a}^{p}\left(r\right)|\Phi_{b}^{s}\left(r^{\prime }\right)\right\rangle$ survives only when *bra* and *ket* are identical determinants (i.e., $\left\langle \Phi_{a}^{p}\left(r\right)|\Phi_{a}^{p}\left(r^{\prime }\right)\right\rangle$) simplifying the calculation of the wavefunction overlap

(24) $$\left\langle \Psi_{i}\left(r\right)|\Psi_{j}\left(r^{\prime }\right)\right\rangle =\sum_{a}^{n}\sum_{p}^{s}C_{\mathrm{ia}}^{p}{C\prime }_{\mathrm{ja}}^{p}\;$$

The presented approach achieves accuracy comparable to the explicit computation of the overlap via Slater determinants (Equation 14), but accelerates immensely the calculation, as will be shown in section 4.2.

### 3.3 Evaluation of excited states transition dipole moment

The probability and intensity of a transition between two electronic states is proportional to the magnitude of the TDM between them. Consequently, in order to evaluate the ESA, it is necessary to compute the TDM between a currently populated excited state and the higher‐lying states. This can be analytically calculated exactly for wavefunction‐based method.^65^ Although this is not possible for the linear response formulation of TD‐DFT, the most used in computational spectroscopy, several developing and applicative works have been done in this direction.^66^, ^67^, ^68^ However, the TDMs can be obtained in an approximate way from the one‐electron transition density matrix between two excited states. The transition density matrix is expressed as:

(25) $$D\left(r,r^{\prime }\right)=\int \Psi^{0}\left(x_{1},x_{2},\ldots ,x_{n}\right)\Psi^{I}\left(x_{1},x_{2},\ldots ,x_{n}\right)dx_{1},dx_{2},\ldots ,dx_{n}\;$$

where Ψ are the wavefunction of the electronic states, **r** the spatial and **x** the spin‐space coordinates. In case of CIS or TD‐DFT, but only if the TDA^64^ is applied to exclude the de‐excitation in the wavefunction expansion, the transition densities can be obtained as diagonal element of this matrix, using the Kohn–Sham molecular orbitals, as

(26) $$D_{m,n}\left(r\right)=\sum_{j}\sum_{k}\omega_{j}\;\phi_{j}\left(r\right)\phi_{k}^{*}\left(r\right)\;$$

In case of a TDA‐TD‐DFT calculations, *ω* corresponds to the element of the vector **X** of the Casida equation.^69^ Multiplying Equation (26) with the dipole moment of each state and integrating over the whole space the TDM is obtained. In our current implementation, this is done by Multiwfn,^70^ which we interfaced to COBRAMM. The interface parses a Gaussian output file to Multiwfn and stores the TDM between the excited states. This interface gives the possibility of using this functionality of the code, but it also represents the starting point of potentially implement other type of wavefunction analysis using Multiwfn in an easy way.

### 3.4 Time‐resolved transient absorption calculation

**FIGURE 2:**
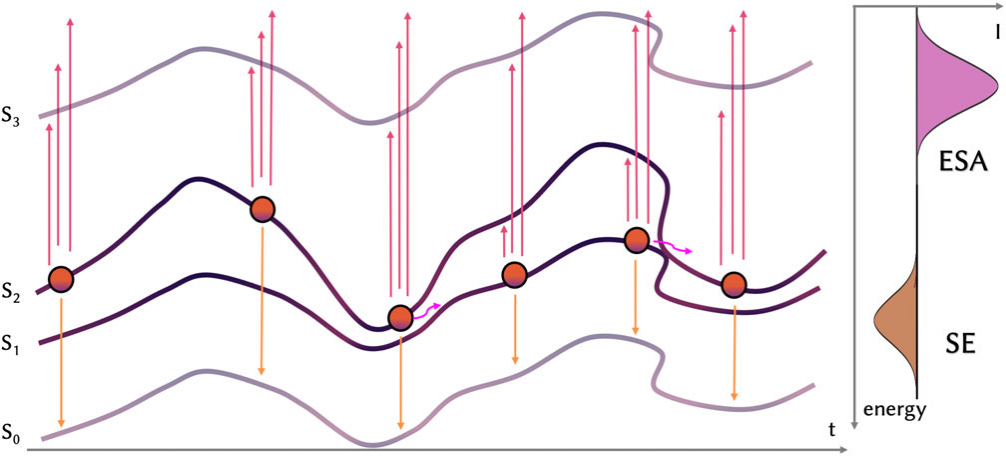
Schematic representation of the workflow to simulate TA spectrum. A trajectory (orange circle) is propagated along the potential energy surface of the active state (*S*_n_) and can hop (pink arrows) between states. For each step (each circle) vertical excitations are run. The SE is convoluted according to the energy and oscillator strength of the *S*_0_ ‐ > Sn transition (yellow arrows and convoluted signal on the right) and the ESA from transitions from Sn to higher states (purple arrows and convoluted signal). ESA, excited state absorption; TA, transient absorption

Once a statistically relevant number of trajectories are propagated, these can be post‐processed to calculate and convolute the time‐resolved TA spectrum by calculating vertical excitations for time steps of the trajectories within a chosen delay time. This might be in principle done on‐the‐fly along the propagation, but the reasons of the post‐process implementation are the following: (i) along the dynamics a small number of excited states are calculated. In order to keep the calculation as light as possible, usually only few states higher in energy, with respect to the initial active one, are computed. However, the ESA can involve states that are not included along the TSH simulation; (ii) the vertical excitations can be calculated at a higher level of theory, for example using a bigger basis set, to obtain more accurate energies; (iii) the excited states absorption can be evaluated multiple times with different level of theory, using the same propagated geometries without re‐running the trajectories. We implemented the full workflow to obtain TA spectrum from an ensemble of trajectory within a python class in COBRAMM, whose functions are called by two auxiliary scripts, one needed to setup and run the vertical excitations, the other to extract TDM and energies and to convolute the PP spectrum. The workflow is schematized in Figure 2. A trajectory is propagated in time and the active state can change after a hop. Within a certain delay time, the current active state at the selected step and the geometry at this specific *t* is extracted, and vertical excitations are computed for the QM part at that geometry, with the inclusion of the point charges in the Hamiltonian. The SE is extracted from the energy and *f* of the transition from the current active state to the ground state and, analogously, the ESA from the active state to the higher‐lying excited states.

In a first step, the spectrum is convoluted from the collection of excitations obtained for each time step and subsequently the convolution in time is applied as explained in section 2. The resulting TA spectrum will show bands of opposite intensities: the ESA will give rise to a positive signal, while the SE will have a negative intensity. The setup script allows to extract the required information from the trajectories' folders and leaves the flexibility to the user to choose functional, basis set, number of excitations and time delay. The analysis script allows convolution with different broadenings and post‐processing tools on the ensemble of the vertical excitations obtained. The default spectrum convolution is the one at the magic angle (section 2.4). Additionally, the user can require the convolution for parallel and orthogonal pulses arrangements and obtain cuts of the spectrum for different times and energies. An additional important feature of the analysis script is the possibility of a single state decomposition of the bands obtained. In this way it is possible to analyze individually and collectively the bands, obtaining crucial insights on their nature. In summary, Table 1 reports all the scripts that automatize the setup, calculation and analysis of the TA spectrum, which are all already available in the current version of COBRAMM software, available from the COBRAMM GitLab repository.

**TABLE 1:** Tasks partition in the new auxiliary scripts of COBRAMM. Alternatively to the setup scripts, the already existing *cobramm.prep* script is still available and under development

Script		Task
cobramm.prep	cobramm‐solvatedchromo.py	Parametrize and solvate the chromophore with a solvent box
	cobramm‐equilibration.py	Minimize, heat and equilibrate the system at MM level
	cobramm‐droplet.py	Create a droplet of solvent surrounding the chromophore, partition the system in H‐M‐L layers and prepares the input for QM/MM optimization
	cobramm‐wignersampling.py	Prepare a distribution of geometries and momenta sampling the normal modes of the chromophore based on a Wigner distribution
	cobramm‐postwignerequilibration.py	Equilibrate the solvent around the geometries obtained by Wigner distribution and prepare the inputs for TSH
cobramm‐plot.py		Store and plot the result of a TSH dynamics
cobramm‐populations.py		Extract the population of the excited states from an ensemble of trajectories
cobramm‐runpumprobe.py		Setup and run vertical excitation along the simulated trajectories
cobramm‐pumprobespectrum.py		Convolute, store and plot the TA spectrum and perform additional post‐processing analysis

Since the full simulation workflow requires many calculations on different steps and levels, we decided to offer individual scripts to setup and run individual tasks. This guarantees a full support throughout the different steps, but leaves the possibility to the user to choose, parametrize, control or restart any part of the workflow individually. That avoids having a full black‐box approach and allows the user to have control over the whole complex workflow.

## 4 EXEMPLARY APPLICATION: DMABN

The excited states properties and dynamics of DMABN have been intensively studied and properly characterized.^71^, ^72^, ^73^, ^74^, ^75^, ^76^ The reason for the interest behind this molecule is the quite unusual dual fluorescence it exhibits, with a second less intense fluorescent band that is temperature and solvent dependent. The photophysics of this molecule can be summarized as the following: a first absorption band is risen in the UV range by population of the S_2_ state, L_a_ in Platt's notation, predicted to be at TD‐DFT level at around 5–5.5 eV. This state is very close in energy with the *L*_b_ state that it is populated in an ultrafast timescale (tens of fs). This state is darker but still emits, producing the first emission band. The nature of the second fluorescent signal was under debate for long, but by now the most accepted theory is that the emission comes for a longer time scale (ps time scale) from a different excited state that involved an intramolecular charge transfer following the twisting of the dimethylamine group. These studies included TR spectroscopical measurements and, very recently, the TR‐PP spectrum in acetonitrile (MeCN) has been characterized computationally and reported in literature.^31^ Finally, DMABN was recently proposed as a molecular model system to test nonadiabatic methods since its *S*_2_/*S*_1_ decay resembles the second one‐dimensional model original proposed by Tully for the same aim, and already used with this purpose.^77^ For all these reasons, DMABN represents an ideal system to test the reliability and robustness of our implementation on its different steps. We will first test our TSH implementation, with the hopping driven by the computation of WFO for the *S*_2_/*S*_1_ early relaxation within the first 100 fs. Second, we will test the setup to calculate the TA spectrum on the same 100 fs long trajectories, to map the very first nonadiabatic event during the dynamic of this molecule.

### 4.1 Computational details

The initial DMABN geometry was solvated with a box of 24 Å of MeCN molecules. The parameters for MeCN were taken from literature,^78^ while the General Amber Force Field was used to parametrize the chromophore.^60^ The whole system was minimized classically for 1000 cycles, half with steepest descent and half with gradient conjugated method, then heated to 300 K for 20 ps and then pressure and volume equilibrated in for 300 ps with the temperature kept constant with a Langevin thermostat.^79^ During all these steps SHAKE was activated to constrain all the bond involving hydrogen atoms,^80^ allowing a time step of 2 fs, periodic boundary conditions were used and the cutoff to calculate the electrostatic interaction in the real space within the particle meshed Ewald scheme was set to 12 Å.^81^ The final equilibration step was used to cutoff a droplet of 16 Å of MeCN molecules surrounding the centered the chromophore. A QM/MM optimization at CAM‐B3LYP/6‐31g*/MM^82^, ^83^ level was performed until convergence, with the H layer including the DMABN only and the M layer including the closest molecules falling within 6 Å. The rest of MeCN molecules in the droplet were kept in the L layer. After the convergence was reached, the frequencies of the H layer were calculated and used to obtain a distribution of geometries and momenta for the QM part by Wigner sampling. For each of these geometries, the M solvent molecules were equilibrated around the QM new geometries, kept frozen, for 50 ps. The same M‐L partition of solvent molecules as for the QM/MM optimization was kept and it has been used for the TSH trajectories as well. These QM/MM setups were used to propagate 150 independent trajectories starting from *S*_2_ for 100 fs with a time step of 0.5 fs. Nonadiabatic couplings were approximated by calculation of wavefunction overlaps with the orbital rotation scheme previously described (section 3.2). Energy‐based decoherence correction was applied to damp the electronic coefficients and the velocities were rescaled after a hop along the velocity vectors. Five roots were calculated at CAM‐B3LYP/6‐31g*/MM level. Once the trajectories were terminated, TA spectrum was obtained, from time zero (when the molecule is excited to *S*_2_ and starts propagating), by calculating 10 roots every fs along each of the trajectories. An artificial broadening of 0.2 eV in the energy domain and 10 fs in the time domain was applied.

### 4.2 DMABN non‐adiabatic dynamics

**FIGURE 3:**
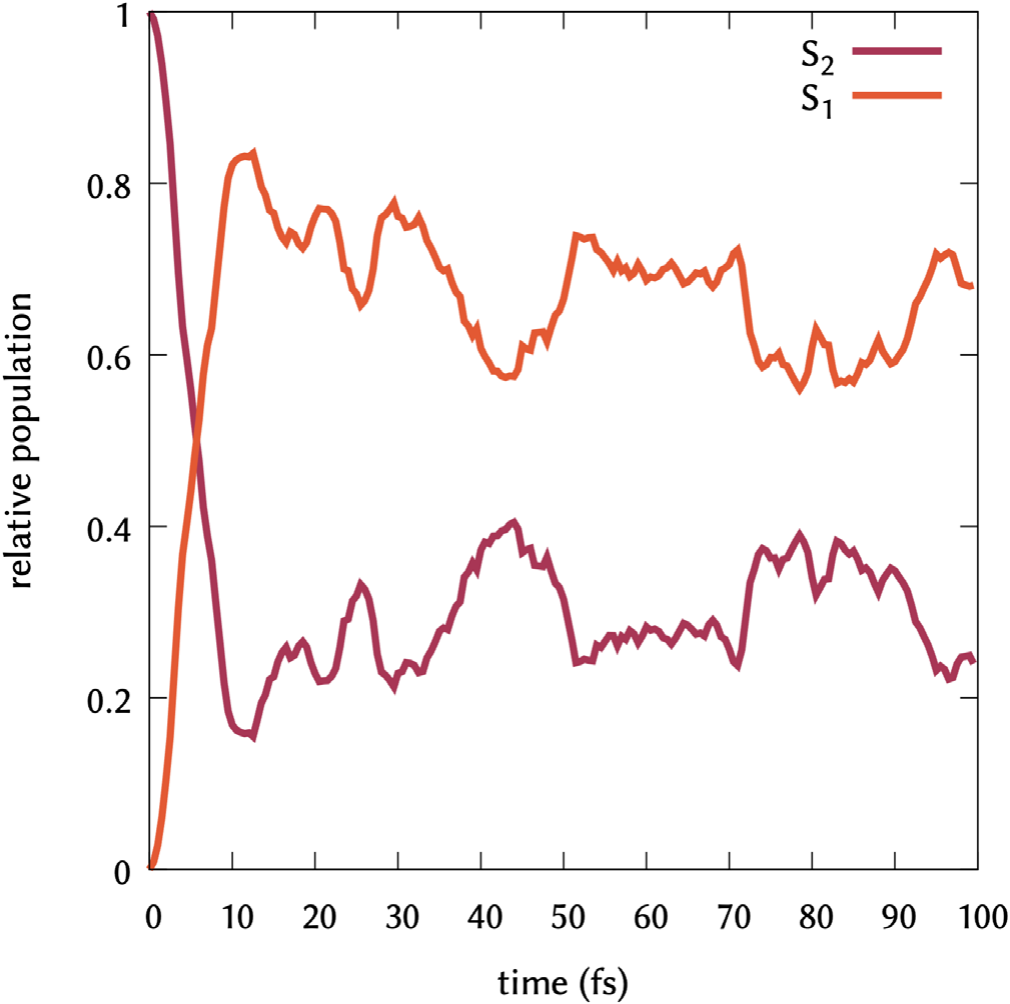
Relative population profile for *S*_2_ (red line) and *S*_1_ (orange line) states averaged over the 150 propagated trajectories

The main feature of the early nonadiabatic dynamics of DMABN is the ultrafast relaxation from *S*_2_ to *S*_1._ ^72^, ^84^ This has been shown to appear within 10–30 fs, depending on method used to describe the dynamics and environment simulated. The *S*_2_/*S*_1_ populations in the first 100 fs obtained for the 150 trajectories ran are shown in Figure 3. Our hopping algorithm, based on built‐in implementation of the calculation of WFN, perfectly reproduces the relaxation within the first 10 fs. Another interesting feature of the dynamics is the multiple crossing of the potential energy surfaces that stay very narrow in energy and allow population transfer back and forth between *S*_1_ and *S*_2_ in the first 100 fs. Previous dynamics with ab initio multiple spawning and TSH in gas phase at TD‐DFT level, showed the oscillations typical of this behavior in the population profiles.^77^ QD simulations on micro‐solvated DMABN in acetonitrile shows the same behavior in the first 100 fs.^73^ TSH dynamics at TD‐DFT level showed a population profile unaffected by the presence of the solvent in the first 100 fs.^85^ However TSH at algebraic diagrammatic construction method showed an almost complete depopulation of *S*_2_, without back hops to that state.^31^ Along our simulation time, we can clearly see the oscillations typical of this behavior in the population profiles, until the *S*_2_ population is 20% of the total one after 100 fs.

**FIGURE 4:**
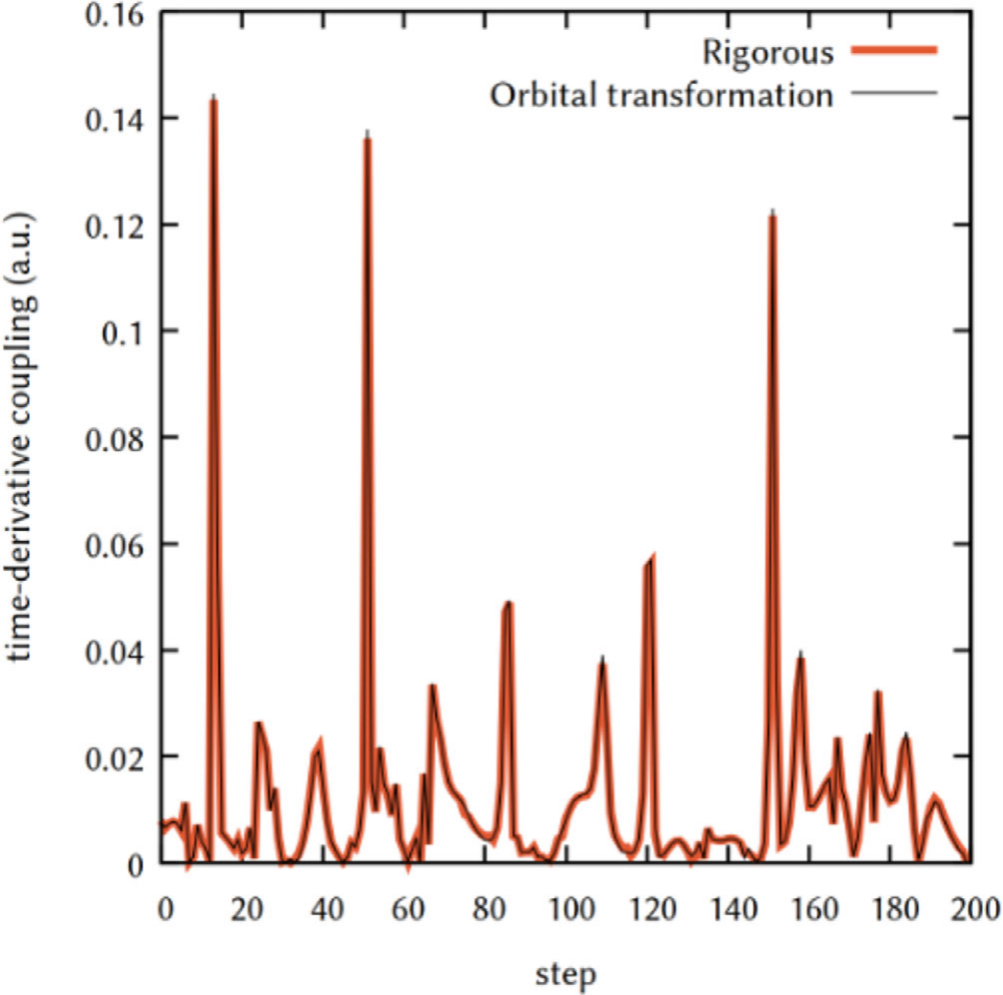
Time‐derivative coupling between *S*_2_ and *S*_1_ along the same adiabatic (no hops allowed) trajectory, starting from the same DMABN/MeCN geometries and momenta, calculated with rigorous formula (bold orange line) and with the routine based on sub‐matrices transformations (thin black line). Time step employed in the simulation was 0.5 fs in both cases.

We tested the performance computing the time derivative coupling by approximating the NAC by WFN with the two algorithms we implemented in COBRAMM (section 3.2). We ran two trajectories starting from the same initial condition and propagate them adiabatically on *S*_2_ surface, forbidding the hops to other states. We computed the time‐derivative coupling between *S*_2_ and *S*_1_ along these trajectories, as show in Figure 4.

As it is visible, the two algorithms give the same result for the WFO, resulting in the same values for the TDC along the trajectories. The main difference stands then in the time needed to compute the WFO. In our implementation, the orbital rotation scheme calculates the TDC in less of a second, for the system used and with the number of roots included, which turned out to be up to 100 times faster along the trajectories studied, suggesting this method as the indicated one, giving identical result in a more efficient way.

### 4.3 DMABN transient absorptium spectrum

**FIGURE 5:**
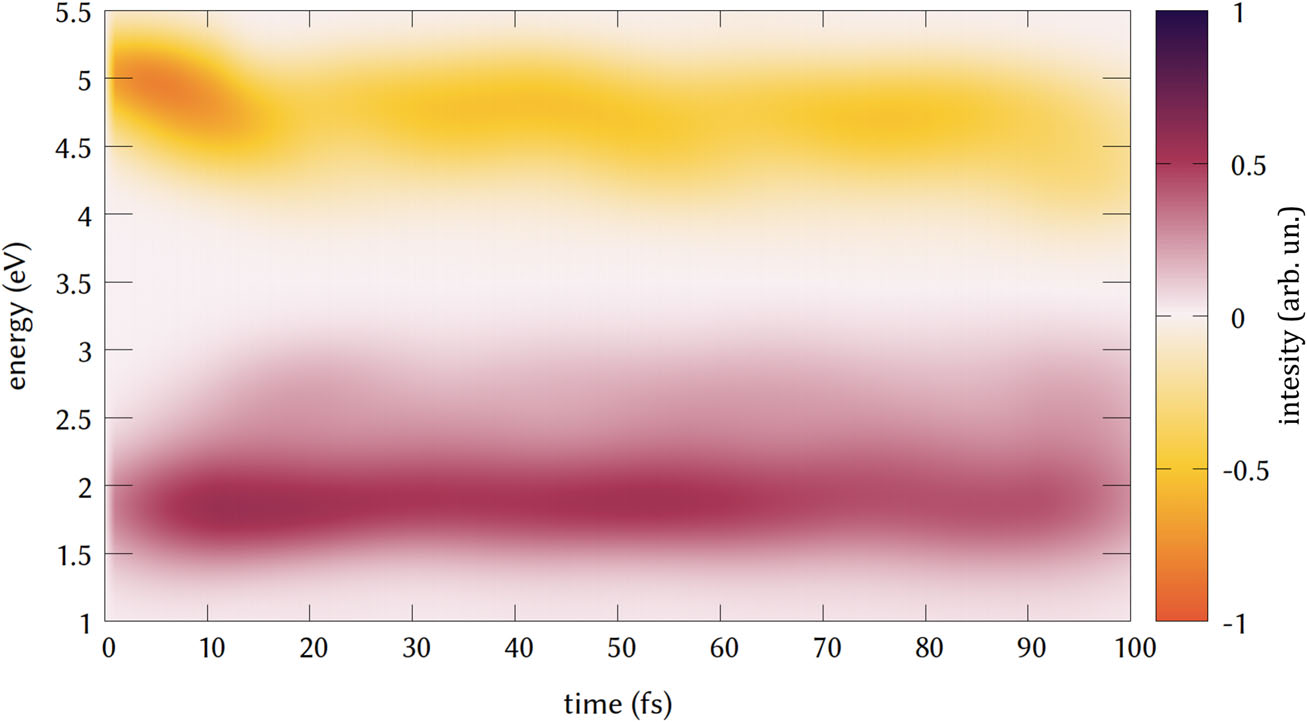
Simulated transient absorption spectrum of DMABN. The signal a lower energy and positive (normalized) intensity is assigned to the excited state absorption (ESA) of the populated states. While the signal rising from 5 eV at negative (normalized) intensity is assigned to the stimulated emission from the populated state to the ground state

The TA spectrum of DMABN has been studied both experimentally and computationally,^31^, ^74^ and helps understanding the ultrafast dynamics of the molecule and the role of the two states involved in the fluorescence of the system. The early spectrum, in the first 100 fs, is composed in the 1–6 eV energy range by two main contributions. At lower energy, around 2.0 eV there is the signal of the ESA, while with opposite sign intensity can be found the SE signal at higher energy. We successfully reproduced the signatures of TA spectrum, which is reported in Figure 5.

The SE signal reflects the ultrafast decay from the bright *S*_2_ state, which is responsible of the intense spot at 5 eV, to the darker *S*_1_ state, that shows the constant band centered at 4.5 eV.

**FIGURE 6:**
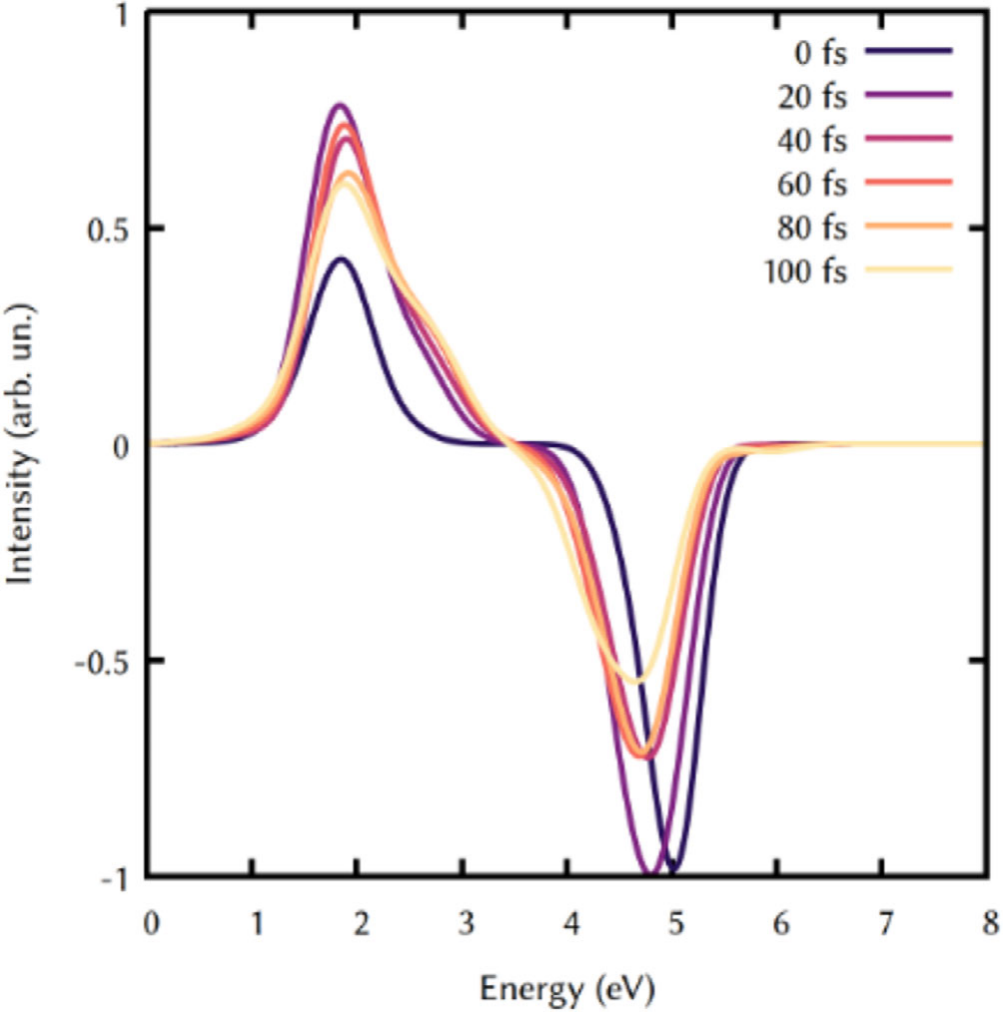
Spectra obtained at different delay times of the probe pulse (interval of 20 fs)

The ESA band is composed by the intense signal centered at 1.75 eV. As for other simulations, we overestimate the initial SE energy.^72^, ^84^ Regarding the ESA, experimentally,^74^ it was found to be centered at around 1.8 eV and slightly higher in the simulations with higher‐accuracy methods (around 2 eV), so we reproduce satisfactorily the energy of the signal. This signal is assigned to the absorption of the local excited state *S*_1_ to higher excited states. The relative intensity of the two signals is also in agreement with previous results, with the strongest signal given by the initial spot of the *S*_2_ emission, and the ESA and SE bands from 10 to 100 fs less intense and constant over this time.^31^, ^72^, ^84^ Both signals are expected to decay at longer time in the picosecond time scale and a signal associated to the emission from a second *S*_1_ minimum is expected to rise, but we stopped our trajectories after 100 fs, since the goal was only to verify and proof the applicability of the workflow implemented and presented, which will be soon applied to simulate the ultrafast TA spectra of other molecules, whose ultrafast dynamics is less clear and might be only simulated at the TD‐DFT level, due to the size of the chromophore. However, the lower computational cost of this protocol would allow at the same time to run longer trajectories with reasonable computational effort. Nonetheless, in the spectrum reported here, the computational flexibility of TD‐DFT allowed us to obtain a convolution using a statistically relevant number of trajectories (150) and vertical excitation (150.000) otherwise not feasible that alleviates the weight of the approximation used. At this point, we can apply the additional post‐processing tools, in order to disentangle the contributions of the transitions to the spectrum. We can first observe the spectrum for different delay times. In particular, we can decompose the spectrum in signals obtained every 20 fs of delay time from the pump and the probe pulse (Figure 6). At time 0 fs, the SE is centered at 5 eV, and it is red shifted and decreases in intensity for longer times. This shows the change in emitting state already after 20 fs.

On the other hand, the ESA signal centered at 1.8 eV shows a relative lower intensity at time 0 fs, where only *S*_2_ is populated, revealing an absorption also from this state. The signal rises in intensity after 20 fs and shows a broadening over 3 eV, indicating the possible role of more than a single transition in the composition of that signal. A similar cut can be done for different energies. In the experiment, this ESA band was found to be centered at 710 nm (1.75 eV),^74^ in excellent agreement with our data, but we cannot directly compare the time evolution of our signal, due to their lower time resolution.

**FIGURE 7:**
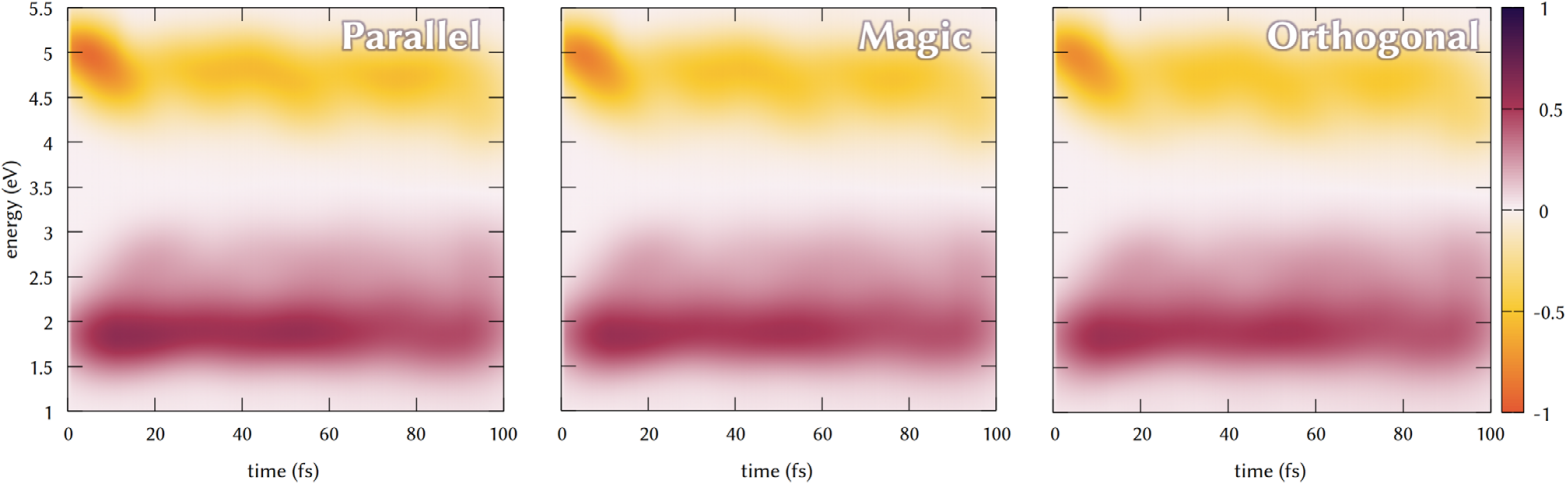
Transient absorption spectra for parallel (left), magic angle (center), and orthogonal (right) orientation of the pump and probe pulses

In Figure 7, we can see the effect of different polarization schemes. In case of parallel arrangement (Figure 7, left) we can notice the intensity of the SE enhanced in the first 10 fs, with respect of the magic angle arrangement (Figure 7, center), and partially along the dynamics.

This is because of the parallel orientation of the *S*_0_ ‐ > *S*_2_ and *S*_2_ ‐ > *S*_0_ TDMs, and that results in a more intense signal at the beginning of the dynamics, in the first 10 fs, and when the population comes back to *S*_2_. The ESA band is enhanced as well in the main signal at 1.75 eV, while in case of orthogonal arrangement, the relative intensity of the blurred band between 2 and 3 eV is enhanced with respect of the main signal, indicating the role of different transitions, which in this case have a TDM orthogonally oriented with respect of the *S*_0_ ‐ > *S*_2_ excitations.

**FIGURE 8:**
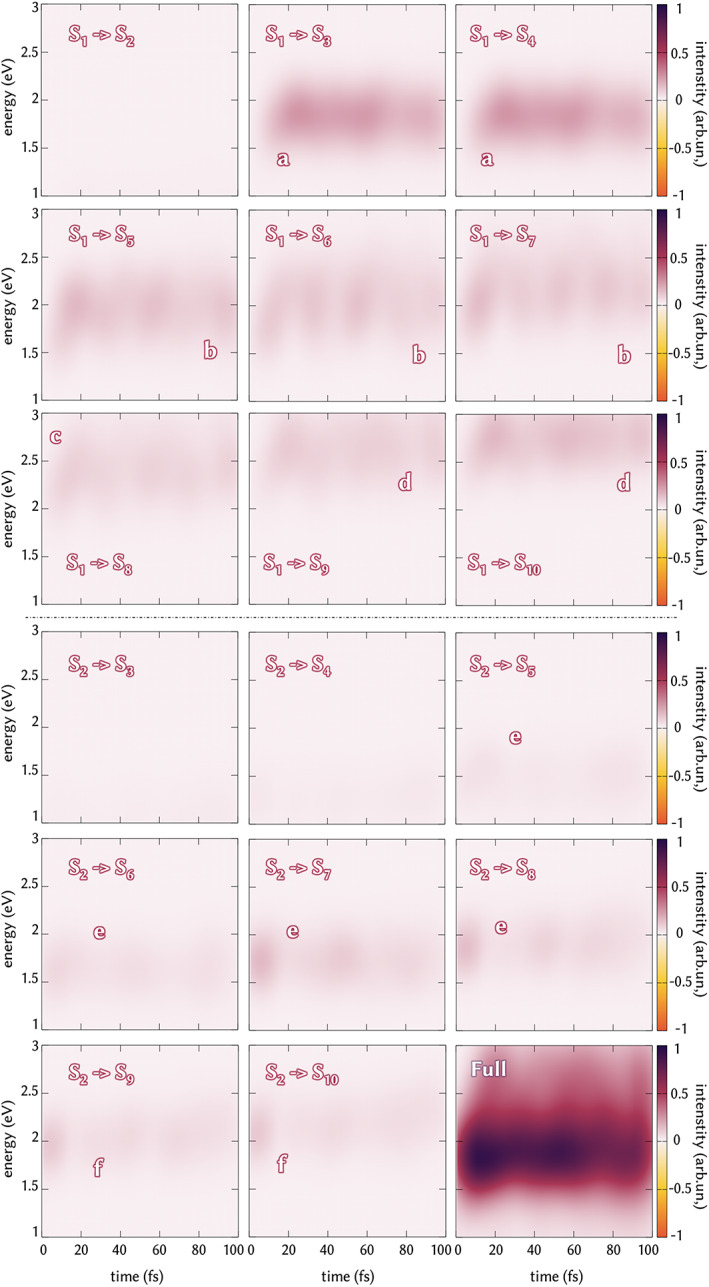
Excited state absorption band decomposition in single contributions of transition from active state *S*_1_ (top half) and *S*_2_ (bottom half) to single *S*_n_ and full signal (bottom right square). Each box represents a single excite state absorption band and the letters indicates individual bands to whose single state absorptions contribute to

In order to finally disentangle the role of different excited states involved in the ESA band, a decomposition of the that band is performed at the magic angle orientation (Figure 8).

We reported the single contributions of the *S*_1_ ‐ > *S*_n_ transition and *S*_2_ ‐ > *S*_n_ transitions. This is practically done by convoluting the spectrum considering only the *S*_1_ (or *S*_2_) to higher states transitions individually, for each step in which the initial state of interest is also active one. We can identify six different sub‐signals (Figure 8a–f) that altogether give rise to the full band (bottom right square, Figure 8). Signals from *S*_1_ start to appear after 10 fs, once this state is populated (Figure 8, top half). In detail, the S_1_ to S_2_ transition does not give any signal in the energy range considered (1–3 eV) due to the close vicinity in energy along the dynamics. The first sub‐signal (a) is centered at 1.75 eV and is obtained from excitations from *S*_1_ to *S*_3_ and *S*_4_. These components are the relatively most intense ones among the whole set of transitions considered and their absorption represents the main contribution to the ESA signal. Transitions from *S*_1_ give rise also to the other part of the band. Excitations to *S*_5_ – *S*_7_ give a weak signal centered at 2 eV (b), while the *S*_1_ ‐ >*S*_8_ absorption is centered at 2.25 eV (c) and finally the upper blurred contribution of the total ESA signal is obtained from transition to *S*_9_ and *S*_10_ (d). Although it is rapidly depopulated, the *S*_2_ relative population along the dynamics still produces some contribution to the total ESA band. Transitions to *S*_3_ and *S*_4_ do not contribute at any time along the dynamics, while a first contribution from this state is found where it is excited to *S*_5_ − *S*_8_ (e), which excitations contribute in minor part to the main signal at 1.75 eV. Additional absorption is found for *S*_2_ – >*S*_9_ and *S*_5_ – >*S*_10,_ which is centered slightly above 2.0 eV. Individual transitions maintain their intensities along the dynamics and confirm the back‐hops between the two excited states along the simulation.

This analysis gives a complete overview on the ESA of DMABN in the first 100 fs, unveiling the main contribution of *S*_1_ – >*S*_3_ and *S*_1_ – >*S*_4_ transitions to the ESA signal, and the relative contributions of the other excited states absorption to the other parts of the total band. The total results show how such type of post‐processing tools might turn out as a crucial tool to disentangle more complex TA signals.

## 5 CONCLUSION AND OUTLOOK

In this work, we presented some of our most recent implementations in COBRAMM, a software developed in our group to simulate photochemistry in complex environment. The new functionalities of the software range from (i) the automatization of the important and delicate task of setup of initial conditions; (ii) the implementation of an internal routine to evaluate fast and efficiently the WFO and compute the TDC; (iii) post‐production tools to analyze the results of TSH simulations; (iv) the evaluation of TDM between excited states at TD‐DFT level; (v) setup, run and convolution of TA spectra resolved in time at the TD‐DFT level; (vi) post‐production analysis including convolution according to different polarization schemes and decomposition of the signal in single transition contributions. Additionally, the efficiency of COBRAMM has been enhanced by rewriting the interfaces for AMBER, for force field‐based calculations, and Gaussian, for TD‐DFT calculations, in an object‐oriented compliant way. The interface with the output and wavefunction analyzer Multiwfn allows the evaluation of the TDM at the TD‐DFT level and opens the opportunities of additional implementations to exploit many of the potentiality of that software, like real‐time excited states characterization along the dynamics, or automatic reparameterization of the chromophore partial charges to account the change in electronic density after promotion and propagation on excited states surfaces. In terms of outlook, the new initial condition preparation toolkits can be additionally increased in flexibility and capabilities to offer an always stronger support to the user in such a delicate task. With this work, we offer for the first time the possibility of simulating TA spectra resolved in time at the TD‐DFT level with a QM/MM scheme, which means, within the limitations of the method, opening the doors of such a type of simulation to medium‐to‐big systems, without losing the effect on the environment, included within the QM/MM formalism, and obtaining a reliable and accurate comparison with experiments, giving insight otherwise impossible to obtain for the ultrafast dynamics of these molecules. In perspective, with all the theoretical considerations of the case, our interface might be adapted to couple different levels of theory for different steps of the protocol, that is, propagating the dynamics on TD‐DFT surfaces and compute the vertical excitations with a multireference wavefunction‐based method, for example in cases where double excitations might be relevant in the TA. We aim to extend the workflow to methods like ADC(2),^86^ for which, being a black box method as TD‐DFT, the only work needed to include it in our workflow would be to write an interface to any QM software where this method is implemented. The same workflow, but not in an automatized way, has been already successfully applied by our group by using multireference methods.^12^ It is our intention to implement an automatization of this workflow, including the choice of active space and other crucial aspects of these kind of calculations. Finally, our protocol could be in the future extended and adapted to simulate other type of TR experiments like TR photoelectron spectroscopy or TR X‐ray absorption spectroscopy.

## Data Availability

Initial conditions and other data needed to reproduce the simulations are available from the corresponding author upon request. The code developed in the framework of this work and the auxiliary scripts presented are available from the COBRAMM GitLab repository (https://gitlab.com/cobrammgroup/cobramm) under the GPLv3 license.

## References

[1] A. H. Zewail , J. Phys. Chem. A 2000, 104(24), 5660.

[2] M. Maiuri , M. Garavelli , G. Cerullo , J. Am. Chem. Soc. 2020, 142(1), 3. 31800225 10.1021/jacs.9b10533

[3] D. Polli , P. Altoè , O. Weingart , K. M. Spillane , C. Manzoni , D. Brida , G. Tomasello , G. Orlandi , P. Kukura , R. A. Mathies , M. Garavelli , G. Cerullo , Nature 2010, 467(7314), 440. 20864998 10.1038/nature09346

[4] T. Kumpulainen , B. Lang , A. Rosspeintner , E. Vauthey , Chem. Rev. 2017, 117(16), 10826. 27957848 10.1021/acs.chemrev.6b00491

[5] J. Woodhouse , G. Nass Kovacs , N. Coquelle , L. M. Uriarte , V. Adam , T. R. M. Barends , M. Byrdin , E. de la Mora , R. Bruce Doak , M. Feliks , M. Field , F. Fieschi , V. Guillon , S. Jakobs , Y. Joti , P. Macheboeuf , K. Motomura , K. Nass , S. Owada , C. M. Roome , C. Ruckebusch , G. Schirò , R. L. Shoeman , M. Thepaut , T. Togashi , K. Tono , M. Yabashi , M. Cammarata , L. Foucar , D. Bourgeois , M. Sliwa , J.‐P. Colletier , I. Schlichting , M. Weik , Nat. Commun. 2020, 11(1), 741. 32029745 10.1038/s41467-020-14537-0 PMC7005145

[6] A. Cannizzo , Phys. Chem. Chem. Phys. 2012, 14(32), 11205. 22781956 10.1039/c2cp40567a

[7] M. Chergui , J. Chem. Phys. 2019, 150(7), 70901. 10.1063/1.5082644 30795676

[8] I. Conti , G. Cerullo , A. Nenov , M. Garavelli , J. Am. Chem. Soc. 2020, 142(38), 16117. 32841559 10.1021/jacs.0c04952 PMC7901644

[9] M. F. Gelin , X. Huang , W. Xie , L. Chen , N. Došlić , W. Domcke , J. Chem. Theory Comput. 2021, 17(4), 2394. 33755464 10.1021/acs.jctc.1c00109

[10] D. Hu , J. Peng , L. Chen , M. F. Gelin , Z. Lan , J. Phys. Chem. Lett. 2021, 12(39), 9710. 34590858 10.1021/acs.jpclett.1c02640

[11] Y.‐C. Shen , J. A. Cina , J. Chem. Phys. 1999, 110(20), 9793.

[12] R. Borrego‐Varillas , A. Nenov , P. Kabaciński , I. Conti , L. Ganzer , A. Oriana , V. K. Jaiswal , I. Delfino , O. Weingart , C. Manzoni , I. Rivalta , M. Garavelli , G. Cerullo , Nat. Commun. 2021, 12(1), 7285. 34907186 10.1038/s41467-021-27535-7 PMC8671501

[13] C. Xu , K. Lin , D. Hu , F. L. Gu , M. F. Gelin , Z. Lan , J. Phys. Chem. Lett. 2022, 13(2), 661. 35023755 10.1021/acs.jpclett.1c03373

[14] T. Piteša , M. Sapunar , A. Ponzi , M. F. Gelin , N. Došlić , W. Domcke , P. Decleva , J. Chem. Theory Comput. 2021, 17(8), 5098. 34269561 10.1021/acs.jctc.1c00396

[15] B. J. Schwartz , P. J. Rossky , J. Chem. Phys. 1994, 101(8), 6917.

[16] Y. Tanimura , S. Mukamel , J. Chem. Phys. 1994, 101(4), 3049.

[17] P. L. McRobbie , E. Geva , J. Phys. Chem. A 2009, 113(39), 10425. 19775171 10.1021/jp905305t

[18] M. Wehrle , M. Šulc , J. Vaníček , Chimia (Aarau). 2011, 65, 334. 21744688 10.2533/chimia.2011.334

[19] M. Šulc , H. Hernández , T. J. Martínez , J. Vaníček , J. Chem. Phys. 2013, 139(3), 34112. 10.1063/1.4813124 23883015

[20] L. Seidner , G. Stock , W. Domcke , J. Chem. Phys. 1995, 103(10), 3998.

[21] D. Keefer , F. Aleotti , J. R. Rouxel , F. Segatta , B. Gu , A. Nenov , M. Garavelli , S. Mukamel , Proc. Natl. Acad. Sci. 2021, 118(3), e2022037118. 33436412 10.1073/pnas.2022037118 PMC7826416

[22] S. Mukamel , Principles of Nonlinear Optical Spectroscopy, Oxford University, Oxford, United Kingdom. 1999.

[23] B. Brüggemann , P. Persson , H.‐D. Meyer , V. May , Chem. Phys. 2008, 347(1), 152.

[24] B. F. E. Curchod , T. J. Martínez , Chem. Rev. 2018, 118(7), 3305. 29465231 10.1021/acs.chemrev.7b00423

[25] S. Dilthey , S. Hahn , G. Stock , J. Chem. Phys. 2000, 112(11), 4910.

[26] H. R. Hudock , B. G. Levine , A. L. Thompson , H. Satzger , D. Townsend , N. Gador , S. Ullrich , A. Stolow , T. J. Martínez , J. Phys. Chem. A 2007, 111(34), 8500. 17685594 10.1021/jp0723665

[27] R. Mitrić , U. Werner , V. Bonačić‐Koutecký , J. Chem. Phys. 2008, 129(16), 164118. 19045258 10.1063/1.3000012

[28] A. Humeniuk , M. Wohlgemuth , T. Suzuki , R. Mitrić , J. Chem. Phys. 2013, 139(13), 134104. 24116549 10.1063/1.4820238

[29] M. Barbatti , WIREs Comput. Mol. Sci. 2011, 1(4), 620.

[30] A. S. Petit , J. E. Subotnik , J. Chem. Phys. 2014, 141(15), 154108. 25338882 10.1063/1.4897258

[31] M. A. Kochman , B. Durbeej , A. Kubas , J. Phys. Chem. A 2021, 125(39), 8635. 34550700 10.1021/acs.jpca.1c06166 PMC8503879

[32] O. Weingart , A. Nenov , P. Altoè , I. Rivalta , J. Segarra‐Martí , I. Dokukina , M. Garavelli , J. Mol. Model. 2018, 24, 271. 30178229 10.1007/s00894-018-3769-6

[33] P. Altoè , M. Stenta , A. Bottoni , M. Garavelli , Theor. Chem. Acc. 2007, 118(1), 219.

[34] H. M. Senn , W. Thiel , Angew. Chemie Int. Ed. 2009, 48(7), 1198. 10.1002/anie.200802019 19173328

[35] M. E. Casida , M. Huix‐Rotllant , Annu. Rev. Phys. Chem. 2012, 63(1), 287. 22242728 10.1146/annurev-physchem-032511-143803

[36] M. J. Field , P. A. Bash , M. Karplus , J. Comput. Chem. 1990, 11, 700.

[37] D. Avagliano , M. Bonfanti , M. Garavelli , L. González , J. Chem. Theory Comput. 2021, 17, 4639. 34114454 10.1021/acs.jctc.1c00318

[38] I. Conti , M. Bonfanti , A. Nenov , I. Rivalta , M. Garavelli , in Photo‐Active Biological Molecular Materials: From Photoinduced Dynamics to Transient Electronic Spectroscopies BT ‐ QM/MM Studies of Light‐Responsive Biological Systems (Eds: T. Andruniów , M. Olivucci ), Springer International Publishing, Cham 2021, p. 77. 10.1007/978-3-030-57721-6_2

[39] G. Karlström , R. Lindh , P.‐Å. Malmqvist , B. O. Roos , U. Ryde , V. Veryazov , P.‐O. Widmark , M. Cossi , B. Schimmelpfennig , P. Neogrady , L. Seijo , Comput. Mater. Sci. 2003, 28(2), 222.

[40] Fdez , I. Galván , M. Vacher , A. Alavi , C. Angeli , F. Aquilante , J. Autschbach , J. J. Bao , S. I. Bokarev , N. A. Bogdanov , R. K. Carlson , L. F. Chibotaru , J. Creutzberg , N. Dattani , M. G. Delcey , S. S. Dong , A. Dreuw , L. Freitag , L. M. Frutos , L. Gagliardi , F. Gendron , A. Giussani , L. González , G. Grell , M. Guo , C. E. Hoyer , M. Johansson , S. Keller , S. Knecht , G. Kovačević , E. Källman , G. Li Manni , M. Lundberg , Y. Ma , S. Mai , J. P. Malhado , P. Å. Malmqvist , P. Marquetand , S. A. Mewes , J. Norell , M. Olivucci , M. Oppel , Q. M. Phung , K. Pierloot , F. Plasser , M. Reiher , A. M. Sand , I. Schapiro , P. Sharma , C. J. Stein , L. K. Sørensen , D. G. Truhlar , M. Ugandi , L. Ungur , A. Valentini , S. Vancoillie , V. Veryazov , O. Weser , T. A. Wesołowski , P.‐O. Widmark , S. Wouters , A. Zech , J. P. Zobel , R. Lindh , J. Chem. Theory Comput. 2019, 15(11), 5925. 31509407 10.1021/acs.jctc.9b00532

[41] H.‐J. Werner , P. J. Knowles , G. Knizia , F. R. Manby , M. Schütz , WIREs Comput. Mol. Sci. 2012, 2, 242.

[42] M. J. Frisch , G. W. Trucks , H. E. Schlegel , G. E. Scuseria , M. A. Robb , J. R. Cheeseman , G. Scalmani , V. Barone , G. A. Petersson , J. B. Foresman , J. D. Fox , Gaussian 16, Gaussian, Wallingford, CT 2016.

[43] R. Salomon‐Ferrer , D. A. Case , R. C. Walker , WIREs Comput. Mol. Sci. 2013, 3(2), 198.

[44] T. Vasilevskaya , W. Thiel , J. Chem. Theory Comput. 2016, 12(8), 3561. 27420296 10.1021/acs.jctc.6b00269

[45] B. Mennucci , S. Corni , Nat. Rev. Chem. 2019, 3(5), 315.

[46] L. W. Chung , W. M. C. Sameera , R. Ramozzi , A. J. Page , M. Hatanaka , G. P. Petrova , T. V. Harris , X. Li , Z. Ke , F. Liu , H.‐B. Li , L. Ding , K. Morokuma , Chem. Rev. 2015, 115(12), 5678. 25853797 10.1021/cr5004419

[47] B. G. Levine , C. Ko , J. Quenneville , T. J. MartÍnez , Mol. Phys. 2006, 104(5–7), 1039.

[48] J. C. Tully , J. Chem. Phys. 1990, 93(2), 1061.

[49] J. C. Tully , R. K. Preston , J. Chem. Phys. 1971, 55(2), 562.

[50] R. Crespo‐Otero , M. Barbatti , Chem. Rev. 2018, 118(15), 7026. 29767966 10.1021/acs.chemrev.7b00577

[51] F. Agostini , B. F. E. Curchod , R. Vuilleumier , I. Tavernelli , E. K. U. Gross , in TDDFT and Quantum‐Classical Dynamics: A Universal Tool Describing the Dynamics of Matter BT—Handbook of Materials Modeling: Methods: Theory and Modeling (Eds: W. Andreoni , S. Yip ), Springer International Publishing, Cham 2018, 1. 10.1007/978-3-319-42913-7_43-1

[52] I. Tavernelli , B. F. E. Curchod , U. Rothlisberger , Phys. Rev. A 2010, 81(5), 52508.

[53] S. Hammes‐Schiffer , J. C. Tully , J. Chem. Phys. 1994, 101(6), 4657.

[54] G. Granucci , M. Persico , J. Chem. Phys. 2007, 126(13), 134114. 17430023 10.1063/1.2715585

[55] L. Verlet , Phys. Rev. 1967, 159(1), 98.

[56] R. M. Hochstrasser , Chem. Phys. 2001, 266(2), 273.

[57] M. Barbatti , K. Sen , Int. J. Quantum Chem. 2016, 116(10), 762.

[58] J. Suchan , D. Hollas , B. F. E. Curchod , P. Slavíček , Faraday Discuss. 2018, 212, 307. 30259011 10.1039/c8fd00088c

[59] D. Avagliano, E. Lorini, L. Gonzàlez, Phil. Trans. R. Soc. A, 2022, 380, 20200381. 10.1098/rsta.2020.0381 PMC8958275 35341304

[60] J. Wang , R. M. Wolf , J. W. Caldwell , P. A. Kollman , D. A. Case , J. Comput. Chem. 2004, 25(9), 1157. 15116359 10.1002/jcc.20035

[61] J. P. Zobel , J. J. Nogueira , L. González , Phys. Chem. Chem. Phys. 2019, 21(26), 13906. 30155549 10.1039/c8cp03273d

[62] I. G. Ryabinkin , J. Nagesh , A. F. Izmaylov , J. Phys. Chem. Lett. 2015, 6(21), 4200. 26538034 10.1021/acs.jpclett.5b02062

[63] F. Plasser , M. Ruckenbauer , S. Mai , M. Oppel , P. Marquetand , L. González , J. Chem. Theory Comput. 2016, 12(3), 1207. 26854874 10.1021/acs.jctc.5b01148 PMC4785508

[64] S. Hirata , M. Head‐Gordon , Chem. Phys. Lett. 1999, 314(3), 291.

[65] P. Cronstrand , O. Christiansen , P. Norman , H. Ågren , Phys. Chem. Chem. Phys. 2000, 2(23), 5357.

[66] X. Sheng , H. Zhu , K. Yin , J. Chen , J. Wang , C. Wang , J. Shao , F. Chen , J. Phys. Chem. C 2020, 124(8), 4693.

[67] D. A. Fedotov , A. C. Paul , H. Koch , F. Santoro , S. Coriani , R. Improta , Phys. Chem. Chem. Phys. 2022, 24, 4987. 35142309 10.1039/d1cp04340d

[68] I. A. Mikhailov , S. Tafur , A. E. Masunov , Phys. Rev. A 2008, 77(1), 12510.

[69] M. E. Casida , Time‐Dependent Density Functional Response Theory for Molecules. in Recent Advances in Density Functional Methods, Vol. 1, World Scientific, Singapore. 1995, p. 155.

[70] T. Lu , F. Chen , J. Comput. Chem. 2012, 33(5), 580. 22162017 10.1002/jcc.22885

[71] M. A. Kochman , A. Tajti , C. A. Morrison , R. J. D. Miller , J. Chem. Theory Comput. 2015, 11(3), 1118. 26579762 10.1021/ct5010609

[72] B. F. E. Curchod , A. Sisto , T. J. Martínez , J. Phys. Chem. A 2017, 121(1), 265. 27976899 10.1021/acs.jpca.6b09962

[73] S. Gómez , E. N. Soysal , G. A. Worth , Molecules 2021, 26, 7247. 10.3390/molecules26237247 PMC8658867 34885829

[74] S. I. Druzhinin , N. P. Ernsting , S. A. Kovalenko , L. P. Lustres , T. A. Senyushkina , K. A. Zachariasse , J. Phys. Chem. A 2006, 110(9), 2955. 16509618 10.1021/jp054496o

[75] I. F. Galván , M. E. Martín , M. A. Aguilar , J. Chem. Theory Comput. 2010, 6(8), 2445. 26613498 10.1021/ct9006713

[76] W. M. Kwok , C. Ma , D. Phillips , P. Matousek , A. W. Parker , M. Towrie , J. Phys. Chem. A 2000, 104(18), 4188.

[77] L. M. Ibele , B. F. E. Curchod , Phys. Chem. Chem. Phys. 2020, 22(27), 15183. 32582887 10.1039/d0cp01353f

[78] X. Grabuleda , C. Jaime , P. A. Kollman , J. Comput. Chem. 2000, 21(10), 901.

[79] J. Liu , D. Li , X. Liu , J. Chem. Phys. 2016, 145(2), 24103. 10.1063/1.4954990 27421393

[80] V. Kräutler , W. F. van Gunsteren , P. H. Hünenberger , J. Comput. Chem. 2001, 22(5), 501.

[81] U. Essmann , L. Perera , M. L. Berkowitz , T. Darden , H. Lee , L. G. Pedersen , J. Chem. Phys. 1995, 103(19), 8577.

[82] T. Yanai , D. P. Tew , N. C. Handy , Chem. Phys. Lett. 2004, 393(1), 51.

[83] R. Ditchfield , W. J. Hehre , J. A. Pople , J. Chem. Phys. 1971, 54(2), 724.

[84] M. A. Kochman , B. Durbeej , J. Phys. Chem. A 2020, 124(11), 2193. 32083861 10.1021/acs.jpca.9b10588

[85] G. R. Medders , E. C. Alguire , A. Jain , J. E. Subotnik , J. Phys. Chem. A 2017, 121(7), 1425. 28098456 10.1021/acs.jpca.6b12120

[86] A. Dreuw , M. Wormit , WIREs Comput. Mol. Sci. 2015, 5(1), 82.

